# Snakebites in Northeastern Brazil: accessing clinical-epidemiological profile as a strategy to deal with Neglected Tropical Diseases

**DOI:** 10.1590/0037-8682-0224-2023

**Published:** 2023-10-06

**Authors:** Sâmia Caroline Melo Araújo, Joseneide Teixeira Câmara, Thaís B. Guedes

**Affiliations:** 1 Universidade Estadual do Maranhão, Programa de Pós-Graduação em Biodiversidade, Ambiente e Saúde, Caxias, MA, Brasil.; 2 Universidade Estadual de Campinas, Instituto de Biologia, Departamento de Biologia Animal, Campinas, SP, Brasil.; 3 Gothenburg Global Biodiversity Center, University of Gothenburg, Department of Biological and Environmental Sciences, Göteborg, Sweden.

**Keywords:** Bothrops, Public Health, Maranhão, Snake antivenom

## Abstract

**Background:**

Brazil ranks first in the number of snakebites in South America. A detailed analysis of these cases is required to improve the public health planning. In this study, we retrospectively examined the clinical and epidemiological profiles of snakebites in Maranhão between January 2009 and December 2019.

**Methods:**

Data were obtained from the compulsory notification forms provided by the Health Department of Maranhão.

**Results:**

A total of 17,658 cases were recorded during the study period. Most of the bites were from snakes belonging to the genus *Bothrops*. Medical care was mostly within three hours after the bite. Most cases were classified as mild and most victims recovered; however, 139 deaths were recorded. Most bites occurred among people aged 20-39 years, mainly among rural workers. The most frequent local clinical manifestations were pain, edema, and ecchymosis. The most common systemic clinical manifestations include neuroparalysis, vagal syndrome, and myolysis. Most snakebites occurred between January and March. The municipalities with the highest number of notifications were Buriticupu (936 cases), Arame (705 cases), and Grajaú (627 cases).

**Conclusions:**

The clinical profile of snakebites in Maranhão is similar to that observed in other states of Northeast Brazil. However, we found that some systemic manifestations are not compatible with the etiology of snakebites, which leads us to believe that the problem could be the lack of knowledge of the health professionals at the site of envenomation, who may not be ready for attendance, and an important lack of health centers with snake antivenom to treat snakebites.

## INTRODUCTION

Snakebites from venomous snakes of medical importance are considered a neglected tropical disease^1^ ^,^ ^2^. Venomous snakebites are an important public health problem because of their frequency, severity, and high morbidity and mortality rates^3^. Additionally, they are associated with poverty, affecting mostly rural workers living in poor conditions in regions of developing countries^1^. Thus, venomous snakebites are also called "disease of poverty" and "occupational disease"^1^ ^,^ ^4^. Snakebite victims suffer from a poor antivenom supply and a shortage of trained health professionals^5^. These factors, added to the under- or non-reporting of snakebites cases, creating a perfect scenario for the extensive gap in the understanding of these injuries on local, regional, and global scales^6^ ^,^ ^7^. The impact of snakebites on human populations has been neglected by either government health agencies or the pharmaceutical industry, reflecting the historical scarcity of programs aimed at minimizing the effects and improving the treatment of this problem globally^8^ ^,^ ^9^. Based on these assumptions, the World Health Organization (WHO) has been implementing a global agenda with several strategies to enable local communities to ensure safe and effective treatment for snakebite victims as well as strengthen the health systems, thus reducing the number of cases by 50% by 2030^8^ ^,^ ^1^.

Approximately 28,000 snakebites caused by venomous snakes of medical importance occur annually in Brazil^10^. However, this number is underestimated because the incidence of snakebites are underreported^11^. Moreover, there is a lack of systematic government planning and adequate training for health professionals to complete compulsory notification forms correctly. Moreover, there are failures in the construction of health education programs regarding the importance of correctly and completely filling out notification forms for snakebites at the national and state levels^11^ ^,^ ^12^. The compulsory notification of snakebites by healthcare centers has been a public policy of collective health in Brazil since 1986^13^, which guides the supply and distribution of antivenoms^14^.

In Brazil, medically important snakes belong to two families, with emphasis on genus *Bothrops*, *Crotalus*, and *Lachesis* belonging to family Viperidae, and *Micrurus* under family Elapidae^15^. At the national level, approximately 90% of snakebites are caused by the genus *Bothrops* ^12^, whose venom cause severe local symptoms such as edema, ecchymosis, and necrosis, as well as systemic symptoms such as cerebrovascular accident (CVA) and acute kidney injury (AKI)^16^ ^,^ ^17^. The genus *Crotalus* ranks second in the number of snakebites, accounting for approximately 8% of the cases^12^. *Crotalus* venom causes local symptoms, such as paresthesia, mild edema, and erythema, as well as systemic symptoms, such as headache, myasthenic facies, myalgia, and renal failure^18^. Bites from snakes of the genus *Lachesis* account for approximately 1% of all cases^19^. *Lachesis* venom causes pain, edema, hemorrhage, and systemic symptoms, including vomiting, hypotension, and abdominal pain^20^. The genus *Micrurus* accounts for approximately 1% of snakebites^19^. *Micrurus* has neurotoxic and myotoxic venom that causes local and systemic symptoms (myasthenic facies, palpebral ptosis, complete limb paralysis, and respiratory paralysis)^21^. The lower limbs are the body regions most affected by venomous snake bites in Brazil, mostly affecting children and male rural workers aged 20 and 59 years old^22^ ^,^ ^23^. Moreover, the incidence of snakebites is influenced by climatic, socioeconomic, and environmental factors^23^ ^,^ ^24^.

In the state of Maranhão, 17 species of medically important snakes have been recorded, corresponding to four genera (*Bothrops*, *Crotalus*, *Lachesis* and *Micrurus*) with wide geographic distributions. The state of Maranhão ranks second in the incidence of venomous snakebites in northeastern Brazil and fourth nationally^10^. However, there have been no detailed studies on the clinical-epidemiological profile of venomous snakebites in this state. Therefore, we aimed to (i) describe the epidemiological and clinical aspects of snakebites caused by venomous snakes of medical importance in Maranhão, (ii) compare the clinical-epidemiological profile of the state with national and global profiles, and (iii) discuss public health strategies that could improve the care of snakebite victims in the state (i.e., train professionals to attend to the victims and correctly fill out the notification form, educational work aimed at the most vulnerable population to minimize the number of snakebites, and strategic organization of the distribution of antivenoms).

## METHODS

### Study area

Of the nine states that comprise the northeastern region of Brazil, Maranhão is the second largest state in terms of territorial extension, covering an area of approximately 330,000 km^2^. The state has 217 municipalities, an estimated population of 6,574,789 inhabitants (63% of which live in urban areas), and a demographic density of 19.81 people per square kilometer^25^. Among the 27 Brazilian Federative Units, Maranhão ranks eighth in the Basic Education Development Index of the public educational system, 25^th^ in the Human Development Index, and 17^th^ in the country's Gross Domestic Product^26^. The state is divided into five mesoregions (north, south, east, west, and center)^26^ and has 18 Regional Health Units covering 4-17 municipalities, of which four do not have a distribution center for antivenom: Zé Doca and Santa Inês Health Regions (in the West Maranhense mesoregion, Viana Health Region (North Maranhense) and São João dos Patos (East Maranhense)^27^. The climate is humid tropical, with mean annual temperatures above 26 ºC and annual rainfall of approximately 700 mm in the central-southern region and over 2,200 mm in the central-northern region^28^. The highest rainfall occurred between January and March^28^. The vegetation mainly consists of two main biomes: Cerrado (east and south) and Amazon (west); however, there is also a mosaic of landscapes, such as restinga, mangroves, Babaçu forest, and ecotonal areas Cerrado-Caatinga and Amazônia-Caatinga^26^. 

### Data collection

We used retrospective data on snakebites caused by venomous snakes of medical importance in Maranhão between January 2009 and December 2019. Data were obtained from compulsory notification forms provided by the Health Department of the State of Maranhão, after approval by the Research Ethical Committee. The epidemiological variables considered were the time of the snakebite (year and month); municipality where the snakebite occurred; the area where the snakebite occurred (urban or rural); location of residence (urban or rural), age, ethnicity (self-defined), sex, and occupational activity of the victim; genus of the snake that bit the victim; anatomical site of the snakebite; time elapsed between the bite and medical care; and severity and evolution of envenomation. The clinical variables considered were local and systemic manifestations. Descriptive results of the clinical and epidemiological profiles are presented through bar and pie charts designed in R version 4.0.3, using the bar plot argument^29^.

### Data analysis

We analyzed the clinico-epidemiological data using descriptive statistical procedures such as absolute frequency (n), relative frequency (%), mean, and standard deviation^30^. The main associations between the classification of envenomation (severe and non-severe [mild and moderate cases]), age group, time between the bite and medical care, and genus of the snake that caused the bite were measured using the odds ratio (OR) with a 95% confidence interval (CI) ^31^ ^,^ ^32^ ^,^ ^33^. We used the chi-square test for the bivariate analysis, and the significance level was set at 5%. We analyzed the data using IBM SPSS® (Statistical Package for the Social Sciences) version 20.0^34^.

### Ethical statement

This study was evaluated, approved, and authorized by the National Research Ethical Committee through the Plataforma Brasil and Ethical Committee of the State University of Maranhão (CAAE 40065720.0.0000.554/2021). 

## RESULTS

**FIGURE 1:**
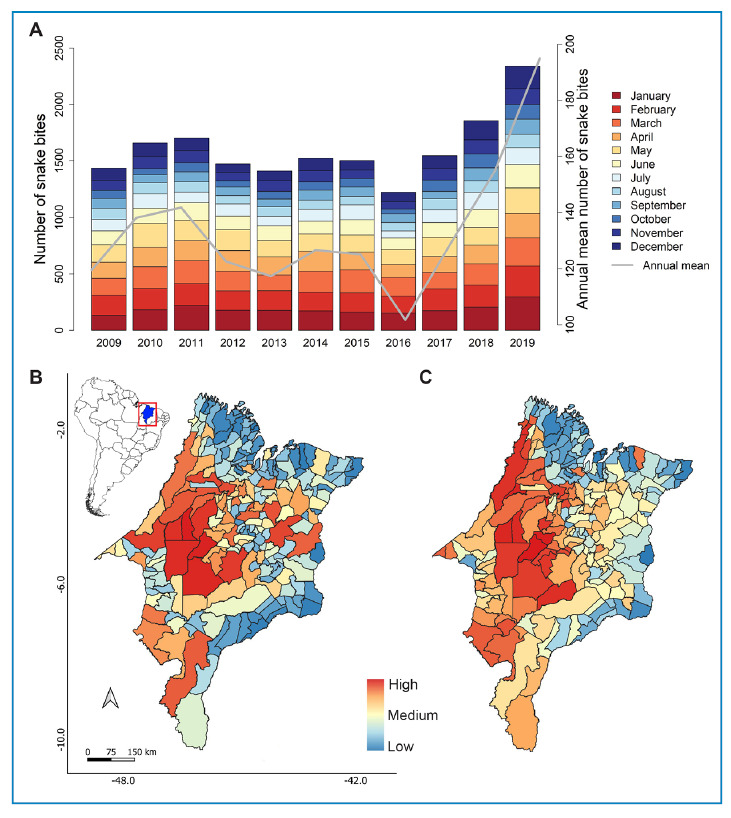
Clinical profile of snakebites from venomous snakes of medical importance in Maranhão, mid-northern region of Brazil, with data from 2009 to 2019. **(A)** Number of snakebites per month and year. **(B)** Number of snakebites for municipalities limits. **(C)** Incidence rates of snakebites per 100,000 inhabitants for municipalities limits.

A total of 17,658 snakebites from venomous snakes of medical importance were reported in Maranhão between 2009 and 2019. The number of venomous snakebites varied over the years, with the highest number reported in 2019, with 2,339 (13%) cases, followed by 2018, with 1,857 (10%) cases (Figure 1A). An increase in the mean annual number of snakebites was observed from 2016 to 2019 (Figure 1A). The months with the highest number of venomous snakebites was from January to March (35%; Figure 1A). October had the lowest number of venomous snakebites cases (5% of total cases; Figure 1A). 

**FIGURE 2:**
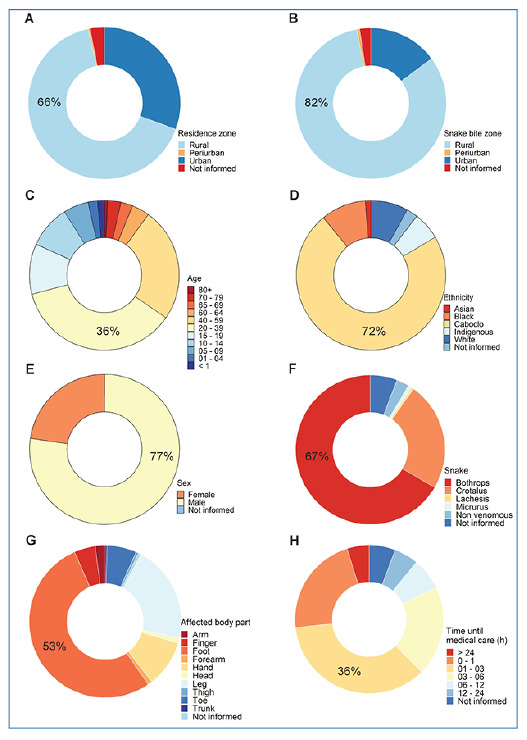
Epidemiological profile of snakebites from venomous snakes of medical importance in Maranhão, mid-northern region of Brazil, with data from 2009 to 2019. **(A)** Zone where the snakebite victims lived. **(B)** Zone where the victims were bitten. **(C)** Age group of snakebite victims. **(D)** Self-defined ethnicities of snakebite victims. **(E)** Sex of snakebite victims. **(F)** Genus of the snake that bit the victims. **(G)** The anatomical regions frequently bitten by snakes. **(H)** Time elapsed between the snakebite and the victims receiving medical care.

The municipalities that reported the highest number of snakebites from venomous snakes of medical importance were Buriticupu at 936 (5%) cases, Arame at 705 (4%) cases, and Grajaú at 627 (3%) cases (Figure 1B). These municipalities are in the western and central mesoregions of Maranhão, Brazil. Regarding the incidence of snakebites per 100,000 inhabitants, the municipalities of Arame (2223.83), Marajá do Sena (1863.12), Tufilândia (1751.25), São João do Carú (1567.96), Bom Jesus das Selvas (1458.24), and Buriticupu (1434.77) (Figure 1C) showed the highest values. Most victims of snakebites (11,650 cases, 66% of the total) resided in rural areas (Figure 2A), and most were bitten while in rural areas (14,497 cases, 82% of the total; Figure 2B). Of the overall snakebite cases, 535 (3%) lacked information on the location of residence of the victims, and 425 (2%) on the geographic area where the snakebite occurred (Figure 2AB ).

The age group most affected by snakebites was 20-39 years with 6,405 cases (36%), followed by the age group 40-59 years with 4,351 cases (25%) (Figure 2C). Regarding self-defined ethnicities, most victims (12,800 cases, 72%) were caboclo people (i.e., Brazilians of mixed white and indigenous or black and indigenous ancestry) (Figure 2D). Males were bitten by snakes more often, with 13,657 (77%) cases (Figure 2E). Regarding occupational activity, 7,913 (45%) victims were rural workers (agriculture and livestock), followed by students with 2,779 (16%) cases. In the notification form, self-defined ethnicity was not reported in 433 (2%) cases, sex in two (0.01%) cases, and occupation in 5,450 (31%) cases.

**FIGURE 3:**
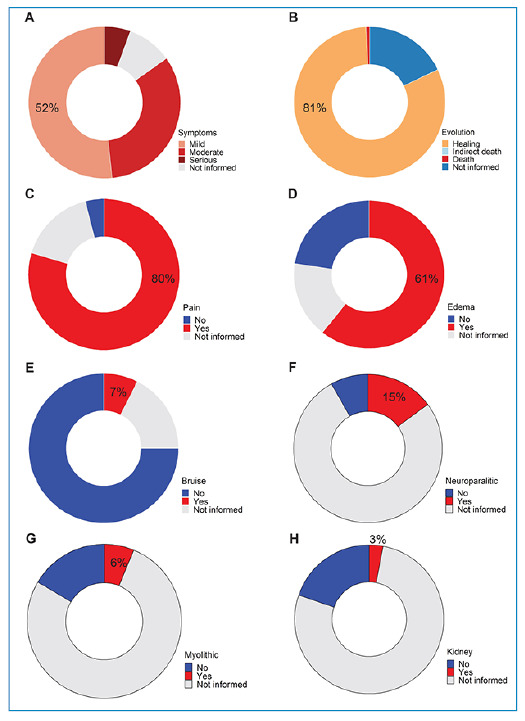
Epidemiological profile of snakebites from venomous snakes of medical importance in Maranhão, mid-northern region of Brazil, with data from 2009 to 2019. **(A)** Classification of symptoms of the snakebite victims. **(B)** Evolution of the case of snakebite victims. Main local clinical manifestations in the victims: **(C)** Pain; **(D)** Edema; **(E)** Ecchymosis or Bruise. Main systemic manifestations in the victims: **(F)** Neuroparalysis; **(G)** Myolythic; **(H)** Renal failure.

Most of the bites were from snakes of the genus *Bothrops* at 11,753 (67%) cases, followed by *Crotalus* at 4,164 (24%) cases, *Micrurus* at 135 (1%) cases, and *Lachesis* at 96 (1%) cases (Figure 2F). The anatomical region most affected by snakes was the feet with 9,338 (53%) cases, followed by the legs with 3,670 (21%) cases (Figure 2G). Notably, 1,754 (10%) bites occurred on the hands (Figure 2G). Most victims received medical care within three hours of being bitten (10,184 cases, 58%) (Figure 2H). Most envenomations were mild (9,111 cases, 52%; Figure 3A), and most victims recovered (14,359, 81%; Figure 3B). In total, 139 victims died (lethality rate, 0.01; Figure 3B). In the notification form, the species of snake that bit the victim was not reported in 1,033 (6%) cases, the anatomical region of the bite in 98 (1%) cases, the time between the bite and medical care in 1,001 (5%) cases, classification of symptoms in 1,700 (10%) cases, and evolution of the envenomation in 3,156 (18%) cases (Figure 2FGH and Figure 3AB).

The main local clinical manifestations were pain in 14,054 (80%) patients (Figure 3C), edema in 10,712 (61%) patients (Figure 3D), and ecchymosis in 1,314 (7%) patients (Figure 3E). The main systemic clinical manifestations were neuroparalysis in 2,627 (15%) patients, (Figure 3F), vagal syndrome in 1,279 (7%) patients ( Supplementary Material), and myolythic in 1,105 (6%) patients (Figure 3G). Renal failure occurred in 530 (3%) patients (Figure 3H). In 8,485 (48%) patients, a coagulation time test was not performed. Among the local manifestations, the following fields were left blank in the form of notifications: pain (2,898 cases, 16%), edema (2,951 cases, 17%), and ecchymosis (3,108 cases, 18%). Among the systemic manifestations, the following were left blank: neuroparalysis (13,584 cases, 77%), vagal syndrome (13,632 cases, 77%), myolysis (13,638 cases, 77%), and renal failure (13,646 cases, 77%). Coagulation time was not reported in 4,037 (23%) cases. Detailed results of the clinical and epidemiological profiles of snakebites caused by venomous snakes are provided in the  Supplementary Material.

The main associations occurred between the severity of envenomation and age, snake genus, and the time elapsed between the bite and medical care (p<0.05, Table 1). Victims aged 51-60 years were more likely to develop severe cases (OR=1.42; 95% CI, 0.79-2.55), followed by victims over 60 years old (OR=1.29; 95% CI, 0.71-2.34). Envenomation by snakes of the genus *Micrurus* showed a higher association with severe cases (OR=4.97; 95% CI, 3.04-8.13), followed by *Crotalus* envenomation (OR=3.31; 95% CI, 2.89-3.80; Table 1). Severe envenomations were associated with time elapsed between the bite and medical attention between 12 and 24h (OR=2.99; 95% CI, 2.28-3.92) and 6-12h (OR=2.94; 95% CI, 2.26-3.82), respectively (Table 1). 

**TABLE 1:** Odds ratio and 95% confidence interval (CI) for the association between the classification of the symptoms in the snakebite victims and the age group, genus of snake, and the time interval victims received medical care after being bitten.

	Classification of the symptoms in the snakebite victims		
Variables	Serious	Not serious	Odds ratio (CI_95%_)
	N (%)	N (%)	
**Age group (Years)**			
< 1	13 (1.3)	218 (1.5)	1
1 a 10	86 (8.8)	1,360 (9.1)	1.06 (0.58 - 1.93)
11 a 20	167 (17.1)	3,152 (21.0)	0.89 (0.49 - 1.59)
21 a 30	170 (17.4)	2,837 (18.9)	1.01 (0.56 - 1.80)
31 a 40	180 (18.4)	2,534 (16.9)	1.19 (0.67 - 2.13)
41 a 50	126 (12.9)	1,966 (13.1)	1.08 (0.60 - 1.94)
51 a 60	131 (13.4)	1,551 (10.4)	1.42 (0.79 - 2.55)
> 60	105 (10.7)	1,362 (9.1)	1.29 (0.71 - 2.34)
**Genus**			
*Bothrops*	436 (46.4)	10,190 (74.1)	1
*Crotalus*	479 (51.0)	3,383 (24.6)	3.31 (2.89 - 3.80)
*Micrurus*	20 (2.1)	94 (0.7)	4.97 (3.04 - 8.13)
*Lachesis*	5 (0.5)	82 (0.6)	1.42 (0.57 - 3.53)
**Time interval for medical care (hours)**			
0 - 1	154 (16.5)	3,293 (23.1)	1
1 - 3	267 (28.5)	5,576 (39.1)	1.02 (0.84 - 1.25)
3 - 6	236 (25.2)	2,963 (20.8)	1.70 (1.38 - 2.10)
6 - 12	84 (9.0)	1,039 (7.3)	1.73 (1.31 - 2.28)
12 - 24	103 (11.0)	749 (5.2)	2.94 (2.26 - 3.82)
24 +	92 (9.8)	658 (4.6)	2.99 (2.28 - 3.92)

## DISCUSSION

Maranhão has the second highest number of reports of venomous snakebites in northeastern Brazil^10^. This study is the first to provide detailed clinico-epidemiological profile of this harm based on a 10-year retrospective data. The profile observed for the state resembled the national and regional profiles^35^ ^,^ ^36^ ^,^ ^37^ ^,^ ^38^ ^,^ ^24^.

During the analyzed period (2009-2019), 17,658 snakebites were reported in Maranhão. However, these records do not reflect the actual situation in the state. Snakebite underreporting is a regional, national, and global problem^39^. The higher number of reports observed in 2019 and 2018 may be related to the considerable increase in agricultural production in Maranhão in recent years^40^. An increase in the number of cases may also reflect improvements in data notification systems. Moreover, other factors (not evaluated here) may account for the higher number of cases, such as ecological (e.g., climate change) and demographic. 

Corroborating previous studies, our analysis showed a positive association between the severity of envenomation and age, with adults over 50 years of age having a higher chance of suffering from severe conditions, probably due to their higher probability of developing renal failure and local necrosis^23^ ^,^ ^41^ ^,^ ^42^. Recently, Maranhão had substantial increase in the number of agricultural workers, reinforcing the classification of snakebites as occupational disease^40^. The frequency of snakebites affecting the lower limbs and hands corroborates the importance of wearing boots/gaiters and gloves to prevent or minimize this harm^1^.

The predominance of bites by *Bothrops* (lanceheads) may be attributed to the high diversity of this genus in Brazil^43^. This genus is widely distributed geographically and inhabits several ecosystems, including degraded environments^44^. Seven species of *Bothrops* are distributed in Maranhão^45^, with most accidents occurring in the western region of the Amazonian and Cerrado areas, coinciding with the distribution of the species. 

The genus *Crotalus* (rattlesnakes) includes species typical of open, arid, and semi-arid areas, but can inhabit savanna enclaves in the Amazon and Atlantic Forest, as well as pasture, disturbed, and urbanized areas^44^ ^,^ ^46^. In the state of Maranhão, most Crotalus acidentes was reported in the eastern region, coinciding with the Cerrado areas where the species inhabit. 

Eight species of *Micrurus* (coral snakes) exists in Maranhão, with a wide geographic distribution^45^; however, most of the envenomations occur in the northeast region of the state. They have fossorial habits, low aggressiveness, short fangs, and small mouth openings, which may explains the low incidence of bites by this genus in other regions of the state. Coral snake envenomation is considered potentially serious because it can lead to severe respiratory failure, even in cases where symptoms appear later^21^ ^,^ ^47^ ^,^ ^48^. 

Snakes of the genus *Lachesis* (bushmasters) are typical found in forest areas, occurring widely in the Amazon and northern Atlantic Forest^44^. In Maranhão, snakebites from *Lachesis* have been documented in areas where snakes had not yet been recorded. Bites from *Lachesis* species are uncommon as they are rare animals, and thus difficult to detect, which predominantly occur in primary forests. Because the symptoms of envenomation caused by this species can be mistaken for those of *Bothrops*, we believe that the reported snakebites from *Lachesis* may have been caused by *Bothrops*.

In some regions (e.g., Roraima State), it is possible to observe differences in the distribution of snakebites by snake genus in different environments (forested and open areas)^49^. A similar case could be observed in the state of Maranhão; however, since the sampling gaps in the geographical distribution of snakes are huge in the state^45^ a proper analysis should be performed to investigate whether this hypothesis is real or an artifact based on sampling gaps of both snake distribution and filling the snakebite forms.

Most snakebite cases were treated within three hours and were classified as mild. Most victims recovered, potentially owing to their awareness of seeking medical care immediately, existence of health units with available antivenoms, and timely assistance from health professionals^38^. A positive association between the classification of cases and the time until treatment has been observed in several studies. This association emphasizes the importance of starting treatment quickly, since the longer the time elapsed between bite and care, the higher the likelihood of severe manifestations and death^50^ ^,^ ^11^. Treatment should be started in parallel with the coagulation time to track venom-induced coagulopathies^2^. The local and systemic clinical manifestations observed in most cases corroborate those of previous studies describing these symptoms in snake envenomations^21^ ^,^ ^20^ ^,^ ^18^.

Venomous snakebites occur mostly during rainy and hot months, when prey abundance and snake activity are higher^5^ ^,^ ^36^ ^,^ ^37^ ^,^ ^38^ ^,^ ^24^. Moreover, the same period was associated with higher agricultural activity, which increased the probability of encounters between snakes and humans^40^. This may explain the higher number of snakebites between January and March in Maranhão, as this was the period with the highest rainfall in the state, and generally, the highest agricultural activity^28^ ^,^ ^51^.

The municipalities with the highest number of notifications and snakebites were located in the western Maranhão and central Maranhão mesoregions. These regions have agricultural production areas^40^ ^,^ ^51^, which may explain the high notification rates, as snakebites mainly affect agricultural workers. In addition, such regions coincide with the "arc of deforestation" zone, corroborating previous studies that show a positive relationship between areas of intensive deforestation and the increase in the number of snakebites^52^. Moreover, these regions are located within the boundaries of Brazil’s Legal Amazon, a highly biodiverse area protected by indigenous territory reserves that do not prevent the advance of deforestation and illegal mining that facilitates encounters between humans and snakes^53^. A recent study characterized these mesoregions as areas of high risk for snakebites, placing them as priority areas for actions aimed at minimizing snakebite injuries^45^. Identifying areas with a high incidence of snakebites is relevant to orientate healthcare efforts focused on the needs and peculiarities of vulnerable populations, thus contributing directly to the strategies proposed by WHO. These municipalities are located in areas unassisted by antivenom centers, leaving victims helpless and without rapid and effective treatment^54^.

In turn, failure to fill out the sections on the notification forms "Not informed (not filled out or keep in blank)" may reflect a lack of understanding by health professionals of the importance of entirely and correctly filling out the forms. This makes it difficult to treat victims and plan the distribution of antivenoms, which depends directly on epidemiological criteria. Moreover, the Ministry of Health is not obliged to fill out some variables in the form (e.g., geographic region of snakebite, time elapsed between the snakebite and treatment, and case evolution), which hinders a more refined analysis of cases^10^. A possible example of errors related to filling out the notification forms is that the most frequent systemic clinical manifestations were neuroparalysis cases, a symptom that cannot be explained or related to envenomation caused by *Bothrops* bites^55^; however, the myolytic manifestations were recorded in 6% of cases, while accidents with *Crotalus* corresponded to 24% of the snakebites. Notably, while looking for data on snakebites caused by venomous snakes of medical importance, we obtained one record (not considered herein) of a severe case of a bite by a non-venomous snake in which the victim developed systemic manifestations and received antivenom. This may be due to taxonomic misidentification by healthcare professionals, highlighting the importance of properly identifying venomous snakes.

## CONCLUSION

Maranhão ranks second in the number of venomous snakebites in northeastern Brazil. This is the first study to provide robust data for epidemiological surveillance to assist snakebite victims. We found that some systemic manifestations were not compatible with the etiology of snakebites, which led us to postulate that the problem could be due to the lack of knowledge of the health professionals who are at the site of envenomation and may not be ready for the attendance. Because snakebites require a holistic understanding of public health, our results highlighted that a short-time action is required to train health professionals in recognizing snakebites from medically important species, promote proper patient care, and correctly fill out the compulsory notification of snakebite forms. 

Compulsory notification of snakebite forms is the basis for antivenom distribution, allowing health centers to be appropriately allocated according to the needs of the region. Similarly, epidemiological surveillance shows that people living in remote areas require a long time to reach treatment centers, and thus resort to alternative inefficient methods, further aggravating the situation. 

## Data Availability

Additional information supporting all results presented in this paper is available in the  Supplementary Materials.

## References

[1] World Health Organization (WHO) (2021). Snakebite envenoming.

[2] Knudsen C, Jürgensen JA, Fons S, Haack AM, Friis RUW, Dam SH (2021). Snakebite Envenoming Diagnosis and Diagnostics. Front Immunol.

[3] Musah Y, Ameade EPK, Attuquayefio DK, Holbech LH (2019). Epidemiology, ecology and human perceptions of snakebites in a savanna community of northern Ghana. PLoS Negl Trop Dis.

[4] Schneider MC, Min K-d, Hamrick PN, Montebello LR, Ranieri TM, Mardini L (2021). Overview of snakebite in Brazil: Possible drivers and a tool for risk mapping. PLoS Negl Trop Dis.

[5] Citeli N, Carvalho M, Carvalho BM, Magalhães MAFM, Bochner R (2020). Bushmaster bites in Brazil: ecological niche modeling and spatial analysis to improve human health measures. Cuad herpetol.

[6] Leynaud GC, Reati GJ (2009). Identificación de las zonas de riesgo ofídico en Córdoba, Argentina, mediante el programa SIGEpi. Rev Panam Salud Publica.

[7] Bisneto PF, Alcântara JA, Silva IM, Sachett JAG, Bernarde PS, Monteiro WM (2019). Coral snake bites in Brazilian Amazonia: perpetrating species, epidemiology and clinical aspects. Toxicon.

[8] Gutiérrez JM, Warrell DA, Williams DJ, Jensen S, Brown N (2013). The Need for Full Integration of Snakebite Envenoming within a Global Strategy to Combat the Neglected Tropical Diseases: The Way Forward. PLOS Neglected Trop Dis.

[9] Gutiérrez JM (2020). Snakebite envenoming from an Ecohealth perspective. Toxicon.

[10] Ministério da Saúde (MS). Secretaria de Vigilância em Saúde (2023). Sistema de Informação de Agravos de Notificação - Sinan Net.

[11] Mise YF, Lira-da-Silva RM, Carvalho FM (2018). Time to treatment and severity of snake envenoming in Brazil. Rev Panam Salud Pública.

[12] Bochner R, Fiszon JT, Machado C (2014). A Profile of Snake Bites in Brazil, 2001 to 2012. J Toxicol Clin Toxicol.

[13] Fan HW, Monteiro WM, Silva AM, Tambourgi DV, Silva IM, Sampaio VS (2015). Snakebites and Scorpion Stings in the Brazilian Amazon: Identifying Research Priorities for a Largely Neglected Problem. PLoS Negl Trop Dis.

[14] Salomão MG, Oliveira PL, Machado C (2018). Epidemiologia dos acidentes por animais peçonhentos e a distribuição de soros: estado de arte e a situação mundial. Rev Salud Pública.

[15] Melgarejo AR, Cardoso JLC, França FOS, Wen FH, Málaque CMS, Haddad V (2009). Animais peçonhentos no Brasil: biologia, clínica e terapêutica.

[16] Sachett JAG, Silva AM, Dantas AWCB, Dantas TR, Colombini M, Silva AMM (2020). Cerebrovascular Accidents Related to Snakebites in the Amazon: Two Case Reports. Wilderness Environ Med.

[17] Oliveira SS, Sampaio VS, Sachett JAG, Alves EC, Silva VC, Lima JAA (2016). Snakebites in the Brazilian Amazon: Current Knowledge and Perspectives. Clin Toxicol.

[18] Azevedo-Marques MM, Hering SE, Cupo P, Cardoso JLC, França FOS, Wen FH, Málaque CMS, Haddad V (2009). Animais peçonhentos no Brasil: biologia, clínica e terapêutica.

[19] Chippaux JP (2015). Epidemiology of envenomations by terrestrial venomous animals in Brazil based on case reporting: from obvious facts to contingencies. J Venom Anim Toxins Incl Trop Dis.

[20] Souza RCG, Cardoso JLC, França FOS, Wen FH, Málaque CMS, Haddad V (2009). Animais peçonhentos no Brasil: biologia, clínica e terapêutica.

[21] Bucaretchi F, De Capitani EM, Hyslop S, Jr. NJ Silva (2016). As cobras-corais do Brasil: Biologia, taxonomia, venenos e envenenamentos.

[22] Santos JA, Santos DL, Silva RLO, Pinheiro RA, Cabral MJS, Santos CB (2020). Epidemiological aspects of snakebite accidents, in the state of Alagoas, in the 2018-2019 biennium. Revista Ambientale.

[23] Mise YF, Lira-da-Silva RM, Carvalho FM (2019). Fatal snakebite envenoming and agricultural work in Brazil: A case - control study. Am J Trop Med Hyg.

[24] Ceron K, Vieira C, Carvalho OS, Carrillo JFC, Alonso J, Santana DJ (2021). Epidemiology of snake envenomation from Mato Grosso do Sul, Brazil. PLOS Neglected Trop Dis.

[25] IBGE - Instituto Brasileiro de Geografia e Estatística (2021). Censo Demográfico 2010.

[26] IBGE - Instituto Brasileiro de Geografia e Estatística (2021). Divisão Geográfica do Estado do Maranhão 2017.

[27] Maranhão (2021). Unidades Regionais de Saúde, 2021.

[28] NUGEO - Núcleo Geoambiental da Universidade Estadual do Maranhão (2021). Laboratório de Meteorologia. 2018..

[29] R Core Team (2021). R: a languange and environment for statistical computing.

[30] Ferreira V (2015). Estatística básica.

[31] Rumel D (1986). "Odds ratio": algumas considerações. Rev Saúde Públ.

[32] Szumilas M (2010). Explaining Odds Ratios. J Can Acad Child Adolesc Psychiatry.

[33] Aguiar P, Nunes B (2013). Odds Ratio: Reflexão sobre a Validade de uma Medida de Referência em Epidemiologia. Acta Med Port.

[34] IBM Corp (2011). IBM SPSS Statistics para Windows.

[35] Leite RS, Targino ITG, Lopes YACF, Barros RM, Vieira AA (2013). Epidemiology of snakebite accidents in the municipalities of the state of Paraíba, Brazil. Ciênc Saúde Colet.

[36] Araújo SCM, Andrade EB (2019). Aspectos epidemiológicos dos acidentes ofídicos ocorridos no estado do Piauí, Nordeste do Brasil, entre os anos de 2003 e 2017. Revista Pesquisa e Ensino em Ciências Exatas e da Natureza.

[37] Matos RR, Ignotti E (2020). Incidência de acidentes ofídicos por gêneros de serpentes nos biomas brasileiros. Ciênc Saúde Colet.

[38] Silva AM, Colombini M, Moura-da-Silva AM, Souza RM, Monteiro WM, Bernarde PS (2020). Epidemiological and clinical aspects of snakebites in the upper Juruá River region, western Brazilian Amazonia. Acta Amaz.

[39] Silva AM, Colombini M, Moura-da-Silva AM, Souza RM, Monteiro WM, Bernarde PS (2019). Ethno-knowledge and attitudes regarding snakebites in the Alto Juruá region, Western Brazilian Amazonia. Toxicon.

[40] Maranhão - Agricultura maranhense (2019). Instituto Maranhense de Estudos Socioeconômicos e Cartográficos - INMESC.

[41] Silva AM, Bernarde PS, Abreu LC (2015). Acidentes com animais peçonhentos no Brasil por sexo e idade. J Hum Growth Dev.

[42] Ribeiro LA, Gadia R, Jorge MT (2008). Comparação entre a epidemiologia do acidente e a clínica do envenenamento por serpentes do gênero Bothrops, em adultos idosos e não idosos. Rev Soc Bras Med Trop.

[43] Guedes TB, Entiauspe-Neto OM, Costa HC (2023). Lista de répteis do Brasil: atualização de 2022. Herpetologia Brasileira.

[44] Nogueira CC, Argôlo AJS, Arzamendia V, Azevedo JA, Barbo FE, Bérnils RS (2019). Atlas of Brazilian Snakes: Verified Point-Locality Maps to Mitigate the Wallacean Shortfall in a Megadiverse Snake Fauna. South Am J Herpetol.

[45] Araújo SCM, Ceron K, Guedes TB (2022). Use of geospatial analyses to address snakebite hotspots in mid-northern Brazil - A direction to health planning in shortfall biodiversity knowledge areas. Toxicon.

[46] Guedes TB, Nogueira C, Marques OAV (2014). Diversity, natural history, and geographic distribution of snakes in the Caatinga, Northeastern Brazil. Zootaxa.

[47] Gutiérrez JM, Lomonte B, Aird S, NJ Silva Jr., NJ Silva Jr. (2016). As cobras-corais do Brasil: Biologia, taxonomia, venenos e envenenamentos.

[48] Bisneto PF, Araújo BS, Pereira HS, Silva IM, Sachett JAG, Bernarde PS (2020). Envenomations by coral snakes in an Amazonian metropolis: Ecological, epidemiological and clinical aspects. Toxicon.

[49] Asato MS, Carbonell CC, Martins AG, Moraes CM, Chávez-Olórtegui C, Gadelha MAC, Pardal PPO (2020). Envenoming by the rattlesnake Crotalus durissus ruruima in the state of Roraima, Brazil. Toxicon.

[50] Bernarde PS (2014). Serpentes peçonhentas e acidentes ofídicos no Brasil.

[51] Maranhão (2019). Perfil da Agricultura Maranhese. Secretaria do Estado da Agricultura, Pecuária e Pesca - SAGRIMA.

[52] León-Núñez LJ, Caremo-Ramos G, Gutiérrez JM (2020). Epidemiology of snakebites in Colombia (2008-2016). Rev. Salud Pública.

[53] Martins MB, Martins MB, Oliveira TG (2011). A Reserva Biológica do Gurupi como instrumento de conservação da natureza na Amazônia Oriental.

[54] Citeli NQK, Cavalcante MM, Magalhães MAFM, Bochner R (2018). Lista dos Polos de Soro para Atendimento de Acidentes Ofídicos no Brasil.

[55] França FOS, Málaque CMS, Cardoso JLC, França SFO, Fan HW, Málaque SCM, Haddad V (2009). Animais peçonhentos no Brasil: biologia, clínica e terapêutica dos acidentes.

