# Supplementary material for: Snakebites in Northeastern Brazil: accessing clinical-epidemiological profile as a strategy to deal with Neglected Tropical Diseases
Source: Rev Soc Bras Med Trop. 2023 Oct 6;56:e0224-2023. doi: 10.1590/0037-8682-0224-2023 (PMC10561396; doi:10.1590/0037-8682-0224-2023)
Supplement: Supplementary file 1 [file 1678-9849-rsbmt-56-e0224-2023-supp1.pdf]

Snakebites in Northeastern Brazil: accessing clinical-epidemiological profile as a strategy to deal with Neglected Tropical Diseases

Supplementary Material

A. Number of snakebites caused by venomous snakes of medical importance per year in the state of Maranhão, mid-northern region of Brazil, between 2009 and 2019. In bold the highest value.

Year	Total	%
2009	1,435	8.1
2010	1,658	9.4
2011	1,701	9.6
2012	1,472	8.3
2013	1,409	8.0
2014	1,520	8.6
2015	1,502	8.5
2016	1,221	6.9
2017	1,544	8.7
2018	1,857	10.5
**2019**	**2,339**	**13.2**

B. Number of snakebites caused by venomous snakes of medical importance per month in the state of Maranhão, mid-northern region of Brazil, between 2009 and 2019. In bold the highest value.

Month	Total	%
January	**2,055**	**11.6**
February	**2,052**	**11.6**
March	**2,014**	**11.4**
April	1,804	10.2
May	1,854	10.5
June	1,506	8.5
July	1,222	6.9
August	993	5.6
September	938	5,3
October	876	5.0
November	1,081	6,1
December	1,263	7.2

C. Number of snakebites caused by venomous snakes of medical importance per municipality and incidence rates per 100,000 inhabitants in the state of Maranhão, mid-northern region of Brazil, between 2009 and 2019. In bold the highest value of each category.

Municipalities	Total	Incidence rates
Açailândia	393	377.71
Afonso Cunha	18	304.83
Água Doce do Maranhão	17	146.79
Alcântara	10	45.76
Aldeias Altas	49	204.58
Altamira do Maranhão	64	578.50
Alto Alegre do Maranhão	36	146.35
Alto Alegre do Pindaré	229	737.35
Alto Parnaíba	54	501.58
Amapá do Maranhão	45	699.74
Amarante do Maranhão	421	1109.88
Anajatuba	31	122.57
Anapurus	24	172.18
Apicum-Açú	12	80.22
Araguanã	110	787.23
Araioses	5	11.76
Arame	**705**	**2223.83**
Arari	114	400.17
Axixá	9	78.90
Bacabal	237	236.97
Bacabeira	29	194.30
Bacuri	10	60.23
Bacurituba	7	132.25
Balsas	259	310.08
Barão de Grajáu	4	22.42
Barra do Corda	401	484.12
Barreirinhas	87	158.38
Bela Vista do Maranhão	71	589.26
Belágua	9	137.95
Benedito Leite	4	73.14
Bequimão	32	157.29
Bernardo do Mearim	9	150.10
Boa Vista do Gurupi	69	868.03
Bom Jardim	263	673.51
Bom Jesus das Selvas	415	**1458.24**
Bom Lugar	12	80.98
Brejo	52	155.88
Brejo de Areia	65	1165.50
Buriti	76	281.35
Buriti Bravo	78	340.63
Buriticupu	**936**	**1434.77**
Buritirana	51	344.97
Cachoeira Grande	15	177.60
Cajapió	16	151.04
Cajari	124	676.19
Campestre do Maranhão	31	231.88
Cândido Mendes	22	118.89
Cantanhede	52	254.30
Capinzal do Norte	33	308.47
Carolina	181	755.46
Carutapera	34	154.50
Caxias	249	160.51
Cedral	2	19.42
Central do Maranhão	6	76.07
Centro do Guilherme	116	923.20
Centro Novo do Maranhão	211	1197.37
Chapadinha	162	220.86
Cidelândia	32	233.90
Codo	345	292.28

Coelho Neto	124	265.24
Colinas	105	268.32
Conceição do Lago-Açú	77	533.39
Coroatá	269	435.80
Cururupu	25	76.56
Davinópolis	23	182.84
Dom Pedro	44	193.99
Duque Bacelar	11	103.30
Esperantinópolis	92	498.59
Estreito	200	558.11
Feira Nova do Maranhão	64	787.60
Fernando Falcão	113	1222.81
Formosa da Serra Negra	114	642.00
Fortaleza dos Nogueiras	24	206.08
Fortuna	25	165.58
Godofredo Viana	11	103.43
Goncalves Dias	60	343.21
Governador Archer	61	597.75
Governador Edison Lobão	44	276.82
Governador Eugenio Barros	41	256.39
Governador Luiz Rocha	23	313.48
Governador Newton Bello	35	293.60
Governador Nunes Freire	121	476.36
Graça Aranha	18	293.16
Grajaú	**627**	1009.78
Guimarães	20	165.55
Humberto de Campos	7	26.73
Icatu	26	103.40
Igarapé do Meio	98	780.88
Igarapé Grande	15	135.86
Imperatriz	169	68.28
Itaipava do Grajaú	140	979.23
Itapecuru Mirim	193	310.74
Itinga do Maranhão	117	470.58
Jatobá	18	211.12
Jenipapo dos Vieiras	179	1159.33
João Lisboa	42	206.07
Joselândia	30	194.39
Junco do Maranhão	51	1268.66
Lago da Pedra	218	473.06
Lago do Junco	38	354.18
Lago dos Rodrigues	21	269.44
Lago Verde	28	181.68
Lagoa do Mato	33	301.81
Lagoa Grande do Maranhão	69	656.08
Lajeado Novo	28	404.45
Lima Campos	61	534.01
Loreto	15	131.69
Luís Domingues	3	46.08
Magalhaes de Almeida	8	45.49
Maracaçumé	46	240.15
Marajá do Sena	150	**1863.12**
Maranhãozinho	52	369.71
Mata Roma	49	323.43
Matinha	9	41.12
Matões	44	141.87
Matões do Norte	34	246.48
Milagres do Maranhão	10	123.18
Mirador	63	308.04
Miranda do Norte	45	184.22
Mirinzal	20	140.67
Monção	316	995.65
Montes Altos	45	478.06
Morros	45	253.05
Nina Rodrigues	55	441.27
Nova Colinas	27	552.71
Nova Iorque	6	130.72

Nova Olinda do Maranhão	141	736.91
Olho d'Agua das Cunhas	25	134.40
Olinda Nova do Maranhão	14	106.21
Paco do Lumiar	13	12.37
Palmeirândia	10	53.29
Paraibano	23	114.41
Parnarama	58	167.70
Passagem Franca	45	256.24
Pastos Bons	13	71.95
Paulino Neves	16	110.20
Paulo Ramos	131	652.42
Pedreiras	47	119.14
Pedro do Rosario	39	171.56
Penalva	47	137.16
Peri Mirim	11	79.69
Peritoró	86	405.64
Pindaré Mirim	100	321.01
Pinheiro	47	60.13
Pio XII	198	899.35
Pirapemas	89	512.05
Poção de Pedras	67	339.96
Porto Franco	68	315.84
Porto Rico do Maranhão	3	49.75
Presidente Dutra	118	263.80
Presidente Juscelino	16	138.64
Presidente Medici	22	345.15
Presidente Sarney	14	81.56
Presidente Vargas	45	419.89
Primeira Cruz	3	21.50
Raposa	3	11.40
Riachão	141	697.71
Ribamar Fiquene	42	573.93
Rosario	13	32.85
Sambaíba	13	236.92
Santa Filomena do Maranhão	25	354.06
Santa Helena	27	69.04
Santa Inês	168	217.39
Santa Luzia	404	545.63
Santa Luzia do Paruá	39	172.23
Santa Quitéria do Maranhão	60	205.54
Santa Rita	23	71.06
Santana do Maranhão	21	180.09
Santo Amaro do Maranhão	9	651.23
Santo Antônio dos Lopes	24	167.97
São Benedito do Rio Preto	60	337.10
São Bento	25	61.37
São Bernardo	30	113.31
São Domingos do Azeitão	9	128.88
São Domingos do Maranhão	45	133.90
São Felix de Balsas	11	233.94
São Francisco do Brejão	30	292.37
São Francisco do Maranhão	10	82.33
São João Batista	128	642.57
São João do Carú	193	**1567.96**
São João do Paraíso	49	453.12
São João do Soter	34	197.24
São João dos Patos	17	68.20
São José de Ribamar	13	7.97
São José dos Basílios	19	253.47
São Luís	79	7.78
São Luís Gonzaga do Maranhão	67	332.46
São Mateus do Maranhão	153	391.37
São Pedro da Água Branca	90	748.25
São Pedro dos Crentes	27	610.17
São Raimundo das Mangabeiras	58	331.92
São Raimundo do Doca Bezerra	53	870.28
São Roberto	53	889.71

São Vicente Ferrer	18	86.28
Satubinha	59	492.08
Senador Alexandre Costa	28	273.01
Senador La Rocque	66	366.71
Serrano do Maranhão	5	45.70
Sítio Novo	88	517.59
Sucupira do Norte	24	229.80
Sucupira do Riachão	16	346.85
Tasso Fragoso	36	461.78
Timbiras	70	250.03
Timon	4	2.57
Trizidela do Vale	34	179.39
Tufilândia	98	**1751.25**
Tuntum	142	362.40
Turiaçú	6	17.68
Turilândia	5	21.89
Tutóia	35	66.30
Urbano Santos	40	162.78
Vargem Grande	139	281.31
Viana	50	101.02
Vila Nova dos Martírios	73	648.43
Vitoria do Mearim	191	611.85
Vitorino Freire	178	562.26
Zé Doca	332	661.71

D. Zone where the snakebite victims lived and where the victims were bitten in the state of Maranhão. mid-northern region of Brazil. between 2009 and 2019. In bold the highest value of each category.

Zone	Residence of victim	Snakebite zone
Rural	**11.650**	**14.497**
Urban	5.403	2.625
Periurban	70	111
Not informed	535	425

E. The age group of snakebite victims in the state of Maranhão. mid-northern region of Brazil. between 2009 and 2019. In bold the highest value.

Age group (Years)	Total	%
<1	257	1
1 - 4	360	2
5 - 9	963	5
10 - 14	1.637	9
15 - 19	1.920	11
20 - 39	**6.405**	**36**
40 - 59	4.351	25
60 - 64	678	4
65 - 69	453	3
70 - 79	511	3
80 +	123	1
Total	17.658	100

F. Self-defined ethnicities of snakebite victims in the state of Maranhão. mid-northern region of Brazil. between 2009 and 2019. In bold the highest value.

Self-defined ethnicities	Total	%
White	1.422	8
Black	1.732	10
Asian	217	1
Caboclo	**12.800**	**72**
Indigenous	1.054	6
Not informed	433	2
Total	17.658	100

G. Sex of snakebite victims in the state of Maranhão. mid-northern region of Brazil. between 2009 and 2019. In bold the highest value.

Sex	Total	%
Male	**13.657**	**77.34**
Female	3.999	22.65
Not informed	2	0.01
Ignorado	2	0
Total	17.658	100

H. Kind of the snake that caused snakebite victims in the state of Maranhão. mid-northern region of Brazil. between 2009 and 2019. Venomous snakes of medical importance in Brazil belongs to the genus *Bothrops*. *Crotalus*. *Micrurus* and *Lachesis*. In bold the highest value.

Kind of the snake	Total	%
Bothrops	**11.753**	**65**
Crotalus	4.164	24
Micrurus	135	0.8
Lachesis	96	0.5
Non venomous	477	3
Not informed	1.033	6
Total	17.658	100

I. The anatomical regions most bitten by snakes in the victims in the state of Maranhão. mid-northern region of Brazil. between 2009 and 2019. In bold the highest value.

Anatomical regions	Total	%
Head	188	1
Arm	377	2
Forearm	170	1
Hand	1.754	10
Finger	793	4
Trunk	67	0
Thigh	97	1
Leg	3.670	21
Feet	**9.338**	**53**
Toe	1.106	6
Not informed	98	1
Total	17.658	100

J. The time interval victims received medical care after being bitten in the state of Maranhão. mid-northern region of Brazil. between 2009 and 2019. In bold the highest value.

Timing (hours)	Total	%
0 - 1	3.872	22
1 - 3	**6.312**	**36**
3 - 6	3.456	20
6 - 12	1.228	7
12 - 24	936	5
24 +	853	5
Not informed	1.001	5
Total	17.658	100

L. Classification of the symptoms in the victims in the state of Maranhão. mid-northern region of Brazil. between 2009 and 2019. In bold the highest value.

Symptoms	Total	%
Light	**9.111**	**52**
Moderate	5.869	33
Serious	978	5
Not informed	1.700	10
Total	17.658	100

M. Evolution of the case of victims of snakebites by venomous snakes of medical importance in the state of Maranhão. mid-northern region of Brazil. between 2009 and 2019. In bold the highest value.

Evolution of the victims of snakbites	Total	%
Healing	**14.359**	**81**
Death	139	1
Indirect death	4	0
Not informed	3.156	18
Total	17.658	100

N. Main local clinical manifestations in the victims in the state of Maranhão. mid-northern region of Brazil. between 2009 and 2019. In bold the highest value of each category.

	Pain (%)	Edema (%)	Ecchymosis (%)
Yes	**14.054 (80)**	**10.712 (61)**	1.314 (7)
No	706 (4)	3.995 (23)	**13.236 (75)**
Not informed	2.898 (16)	2951 (16)	3.108 (16)
Total	17.658	17.658	17.658

O. Main systemic manifestations in the victims in the state of Maranhão. mid-northern region of Brazil. between 2009 and 2019. In bold the highest value of each category.

	Neuroparalysis (%)	Vagal syndrome (%)	Myolythic (%)	Renal failure (%)
Yes	2.627 (15)	1.279 (7)	1.105 (6)	530
No	1.447 (8)	2.747 (16)	2.915 (17)	3.482
Not informed	**13.584 (77)**	**13.632 (78)**	**13.638 (78)**	**13.646 (78)**
Total	17.658	17.658	17.658	17.658
